# Metabolic syndrome and risk of incident diabetes: findings from the European Prospective Investigation into Cancer and Nutrition-Potsdam Study

**DOI:** 10.1186/1475-2840-7-35

**Published:** 2008-12-12

**Authors:** Earl S Ford, Matthias B Schulze, Tobias Pischon, Manuela M Bergmann, Hans-Georg Joost, Heiner Boeing

**Affiliations:** 1 Division of Adult and Community Health, National Center for Chronic Disease Prevention and Health Promotion, Centers for Disease Control and Prevention, Atlanta, Georgia, USA; 2 Public Health Nutrition Unit, Technische Universität München, Center of Life and Food Sciences, Freising, Germany; 3 Department of Epidemiology, German Institute for Human Nutrition (DIfE), Potsdam-Rehbrücke, Nuthetal, Germany; 4 Department of Pharmacology, German Institute of Human Nutrition Potsdam-Rehbruecke, Nuthetal, Germany

## Abstract

**Background:**

Several aspects concerning the relationship between the metabolic syndrome and incident diabetes are incompletely understood including the magnitude of the risk estimate, potential gender differences in the associations between the metabolic syndrome and incident diabetes, the associations between the components of the metabolic syndrome and incident diabetes, and whether the metabolic syndrome provides additional prediction beyond its components. To shed light on these issues, we examined the prospective association between the metabolic syndrome defined by the National Cholesterol Education Program (NCEP) and International Diabetes Federation (IDF) and diabetes.

**Methods:**

We used data for 2796 men and women aged 35–65 years from the European Prospective Investigation into Cancer and Nutrition-Potsdam Study followed for an average of 6.9 years. This analysis employed a case-cohort design that included 697 participants who developed diabetes and 2099 participants who did not. Incident diabetes was identified on the basis of self-reports and verified by contacting the patient's attending physician.

**Results:**

The adjusted hazard ratio for the NCEP definition was 4.62 (95% confidence interval [CI]: 3.90–5.48) and that for the IDF definition was 4.59 (95% CI: 3.84–5.50). The adjusted hazard ratios for the NCEP but not IDF definition were higher for women than men. When participants who had no cardiometabolic abnormalities were used as the reference group for the NCEP definition, the adjusted hazard ratio for having 3 or more abnormalities increased to 22.50 (95% CI: 11.21–45.19). Of the five components, abdominal obesity and hyperglycemia were most strongly associated with incident diabetes.

**Conclusion:**

In this study population, both definitions of the metabolic syndrome provided similar estimates of relative risk for incident diabetes. The increase in risk for participants with the metabolic syndrome according to the NCEP definition was very large when contrasted with the risk among those who had no cardiometabolic abnormalities.

## Introduction

The concept of the metabolic syndrome can be traced back to as early as 1923 when an association between hypertension, uric acid, and hyperglycemia was reported [1]. Since major organizations started formulating definitions for this syndrome in 1998, it has been the object of intense research. Although it has been shown to be a significant predictor of diabetes, cardiovascular disease, and all-cause mortality [2], controversy about its importance as a risk factor remains [3].

The most recent definition of the metabolic syndrome was developed by the International Diabetes Federation (IDF) in 2005 [4]. This definition places a major emphasis on central obesity. Since that time, several reports have examined the associations between the metabolic syndrome as defined by the IDF and incident diabetes and compared these risk estimates to those calculated using other definitions [5-13]. However, several issues remain unresolved including the presence of potential gender differences in the risk for incident diabetes associated with the metabolic syndrome and whether the metabolic syndrome offers additional prediction beyond its components. To examine these issues and to compare the ability of the metabolic syndrome to predict the risk of developing diabetes using two definitions, namely those of the National Cholesterol Education Program (NCEP) and IDF, we used data from a large prospective German study.

## Methods

### Study population

The European Prospective Investigation into Cancer and Nutrition (EPIC) Potsdam study is part of the multi-centre prospective cohort study EPIC [14,15]. In Potsdam, Germany, 27,548 subjects, 16,644 women mainly aged 35–65 years and 10,904 men mainly aged 40–65 years, from the general population were recruited between 1994 and 1998 [16]. The baseline examination included anthropometric measurements, a personal interview including questions on prevalent diseases, and a questionnaire on socio-demographic and lifestyle characteristics. Follow-up questionnaires to identify incident cases of diabetes mellitus have been administered every 2 to 3 years. Response rates for each of the three waves of follow-up were about 95%. We also considered questionnaires that were part of the ongoing fourth wave of follow-up round and were sent out until January 31st 2005. By August 31st 2005, 90% of them were returned. Consent was obtained from all participants of the study, and approval was given by the Ethical Committee of the State of Brandenburg, Germany. The conduct of the study was performed in accordance with principles of the Declaration of Helsinki.

### Ascertainment of incident type 2 diabetes

Potentially incident cases of diabetes were those with self-reports of a diabetes diagnosis, diabetes-relevant medication, or dietary treatment due to diabetes. All potentially incident cases were verified by questionnaires mailed to the diagnosing physician asking about the date and type of diagnosis, diagnostic tests, and treatment of diabetes. Only cases with a physician diagnosis of type 2 diabetes (International Classification of Diseases, 10^th ^Revision: E11) and a diagnosis date after the baseline examination were considered as confirmed incident cases of type 2 diabetes and were used in the analysis.

### Ascertainment of prevalent type 2 diabetes

Self-reported diabetes mellitus at baseline was evaluated by a study physician using information on self-reported medical diagnoses, medication records and dieting behavior. Uncertainties regarding a proper diagnosis were clarified with the participant or treating physician. We also used plasma concentrations of glucose to define prevalent diabetes. Because many participants did not provide fasting blood samples, we defined diabetes as a fasting plasma glucose ≥ 126 mg/dl or a nonfasting plasma glucose of ≥ 200 mg/dl.

### Case-cohort construction

The case-cohort consisted of a random sample of participants from the full cohort and all participants from the full cohort who developed incident diabetes. Thus, a random sample of 2,500 participants (subcohort) was drawn from the participants of the full cohort who had blood samples available (26,444 of 27,548). After excluding participants with prevalent diabetes and missing information for study covariates, the subcohort included 2,165 participants. Of the 801 participants from the full cohort with blood samples who developed incident diabetes, 697 remained for analyses after the exclusion criteria were applied. Because the subcohort is representative of the full cohort at baseline in case-cohort studies, the random sample of the full cohort included 66 of the 697 subjects who developed incident type 2 diabetes during follow-up.

### Metabolic syndrome

According to the IDF definition, someone has the metabolic syndrome if he or she has central adiposity plus ≥ 2 of the following four factors [4]:

1. raised concentration of triglycerides: ≥ 150 mg/dl (1.7 mmol/L) or specific treatment for this lipid abnormality;

2. reduced concentration of high-density lipoprotein cholesterol: < 40 mg/dl (1.03 mmol/L) in males and < 50 mg/dl (1.29 mmol/L) in females or specific treatment for this lipid abnormality;

3. raised blood pressure: systolic blood pressure ≥ 130 mmHg or diastolic blood pressure ≥ 85 mmHg or treatment of previously diagnosed hypertension;

4. raised fasting plasma glucose ≥ 100 mg/dl (5.6 mmol/l) or previously diagnosed type 2 diabetes.

For this study of German participants, we used a waist circumference threshold of ≥ 94 cm for men and ≥ 80 cm for women.

Using the 2004 National Heart, Lung, and Blood Institute/American Heart Association revision of the original NCEP criteria, participants who had three or more of the following criteria were defined as having the metabolic syndrome [17,18]: 1. abdominal obesity (waist circumference >102 cm in men and >88 cm in women); 2. concentration of triglycerides ≥ 150 mg/dl (1.7 mmol/l); 3. concentration of high-density lipoprotein cholesterol <40 mg/dl (1.03 mmol/l) in men and <50 mg/dl (1.29 mmol/l) in women; 4. a systolic blood pressure > = 130 mmHg or a diastolic blood pressure ≥ 85 mmHg); and 5. fasting glucose ≥ 100 mg/dl (5.6 mmol/l). The participants who currently reported using antihypertensive were counted as having high blood pressure.

Anthropometric measurement procedures followed standard protocols under strict quality control [19,20]. Three measurements of systolic blood pressure and diastolic blood pressure from participants in the sitting position were recorded. The average of the last two readings was used. Concentrations of triglycerides, high-density lipoprotein cholesterol, and glucose were measured using the ADVIA 1650 chemistry system (Siemens Medical Solutions, Erlangen, Germany).

### Covariates

We included the following covariates from the baseline data collection: age, sex, educational attainment, occupational activity, physical activity level, alcohol consumption, and concentrations of total cholesterol and C-reactive protein. Information on educational attainment, smoking, occupational activity, and physical activity were assessed with a self-administered questionnaire and a personal interview. For physical activity, we considered participation in sports and bicycling, both calculated as the average time spent per week during the 12 months before baseline recruitment. Alcohol use was assessed as part of the food frequency questionnaire. Concentrations of total cholesterol and C-reactive protein, and glucose were measured using the ADVIA 1650 chemistry system (Siemens Medical Solutions, Erlangen, Germany).

### Statistical analyses

The agreement in the classification of the metabolic syndrome using the two definitions was done with the kappa statistic. Differences in baseline characteristics by incident diabetes status were tested with Kruskal-Wallis tests for continuous variables and with Fisher's exact tests or chi-square tests for categorical variables. Cox proportional hazards analysis, using Prentice's pseudo-likelihood approach in the computations [21], was used to estimate hazard ratios and 95% confidence intervals. Age was used as the primary time dependent variable in all models, with entry time defined as the subject's age at recruitment and exit time as the date of diagnosis of diabetes, death, or return of the last follow-up questionnaire. Analyses were adjusted for baseline information including sex, educational attainment (in or no training, vocational training, technical school, technical college or university degree), smoking (never, past, current <20 cigarettes/d, current ≥ 20 cigarettes/d), occupational activity (light, moderate, heavy), physical activity (continuous as h/week), alcohol intake (<0.1 g/d, 0.1–5.0 g/d, 5.1–10.0 g/d, 10.1–20.0 g/d, 20.1–40.0 g/d, >40.0 g/d), and concentrations of total cholesterol (continuous) and C-reactive protein (continuous). All analyses were performed with SAS release 9.1 (SAS Institute, Cary, NC).

## Results

Our final sample included 1186 men (397 events) and 1610 women (300 events). The mean and median follow-up times were 6.9 and 6.5 years, respectively. The prevalence of the metabolic syndrome according to the NCEP definition in the subcohort was 22.5% among all participants, 29.1% in men, and 18.5% in women. The prevalence of the metabolic syndrome according to the IDF definition in the subcohort was 28.3% among all participants, 33.2% in men, and 25.2% in women. The percent agreement between the two definitions was 87.4% (kappa = 0.67, 95% confidence interval [CI]: 0.63, 0.70) for all participants, 83% (kappa = 0.60, 95% CI: 0.54, 0.66) in men, and 90.2% (kappa = 0.72, 95% CI: 0.67, 0.76) in women.

At baseline, participants of the subcohort who developed diabetes were significantly more likely to be older, to have a larger waist circumference, higher systolic blood pressure, higher concentrations of total cholesterol, triglycerides, glucose, and C-reactive protein, and lower concentrations of high-density lipoprotein cholesterol than participants who did not develop diabetes (Table 1). Furthermore, participants who developed diabetes were far more likely to have had the metabolic syndrome at baseline than those who did not develop diabetes. Levels of physical activity, the percentage of participants who had never smoked, and the percentage of participants who consumed ≥ 40 grams of alcohol per day were not significantly different between the two groups.

**Table 1:** Selected characteristics for the subcohort, by incident diabetes status, European Prospective Investigation into Cancer and Nutrition-Potsdam 1994–1998 to 2005

	**Incident diabetes mellitus**				
		
	**No (N = 2099)**		**Yes (N = 66)**		
			
	**Mean or %**	**SD**	**Mean or %**	**SD**	**p**
**Age (years)**	49.5	8.8	55.9	7.0	<0.001
**Waist circumference (cm)**	85.0	12.4	98.6	11.2	<0.001
**Total cholesterol (mg/dl)**	173.3	37.4	183.3	36.3	0.028
**Triglycerides (mg/dl)**	110.5	74.9	179.8	154.4	<0.001
**High-density lipoprotein cholesterol (mg/dl)**	48.1	13.0	40.3	10.6	<0.001
**Systolic blood pressure (mm Hg)**	128.6	17.4	143.8	21.2	<0.001
**Glucose (mg/dl)**	86.9	15.0	103.4	21.5	<0.001
**C-reactive protein (mg/l)**	1.6	2.7	4.4	7.3	<0.001
**Physical activity (h/w)**	6.1	5.8	5.6	6.0	0.307
					
**%, Female**	62.4	--	50.0	--	0.053
**%, Heavy occupational activity**	6.1	--	0.0	--	0.031
**%, Never smokers**	47.7	--	39.4	--	0.211
**%, Alcohol use >40 g/d**	8.3	--	10.6	--	0.497
**Number of NCEP components**					<0.001
0	19.6	--	0.0	--	
1–2	59.6	--	24.2	--	
≥ 3	20.8	--	75.8	--	
**%, Metabolic syndrome (NCEP)**	20.8	--	75.8	--	<0.001
**%, Metabolic syndrome (IDF)**	26.8	--	74.2	--	<0.001

IDF = International Diabetes Federation, NCEP = National Cholesterol Education Program, SD = standard deviation

Both definitions were significant predictors of incident diabetes (Table 2). The adjusted hazard ratio for the NCEP definition was 4.62 (95% confidence interval [CI]: 3.90, 5.48) and that for the IDF definition was 4.59 (95% CI: 3.84, 5.50). When we limited the analyses to the subsample with fasting blood specimens (N = 788, 189 events), the adjusted hazard ratios were 4.83 (95% CI: 3.46, 6.75) for the NCEP definition and 5.62 (95% CI: 3.99, 7.92) for the IDF definition. The hazard ratios for women were larger than those for men only for the NCEP definition (p interaction for sex and metabolic syndrome = 0.001 for NCEP and 0.325 for IDF).

**Table 2:** Adjusted hazard ratios (95% confidence interval) for incident diabetes by status of the metabolic syndrome among participants aged 35–65 years, European Prospective Investigation into Cancer and Nutrition-Potsdam Study 194–1998 to 2005

**Definition of metabolic syndrome**	**Hazard ratio (95% CI)**
**National Cholesterol Education Program**	
Total (697 events, 2796 participants)	4.622 (3.895, 5.484)
Men (397 events, 1186 participants)	3.687 (2.943, 4.619)
Women (300 events, 1610 participants)	6.075 (4.646, 7.944)
	
**International Diabetes Federation**	
Total (697 events, 2796 participants)	4.593 (3.838, 5.498)
Men (397 events, 1186 participants)	4.279 (3.370, 5.434)
Women (300 events, 1610 participants)	4.785 (3.615, 6.333)

Adjusted for age, sex, educational status, smoking status, alcohol use, physical activity, and concentrations of total cholesterol and C-reactive protein.

**Figure 1:**
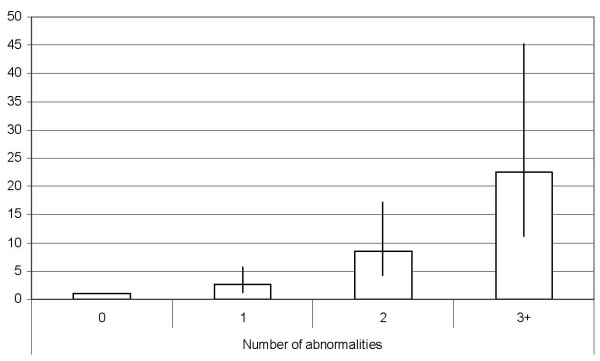
**Hazard ratios and 95% confidence intervals for incident diabetes among 2796 participants aged 35–65 years, by number of cardiometabolic abnormalities, EPIC-Potsdam 1994–1998 to 2005**. Hazard ratios are adjusted for age, sex, educational status, smoking status, alcohol use, occupational activity, physical activity, concentrations of total cholesterol and C-reactive protein, and other components of the metabolic syndrome.

For the NCEP definition, we also investigated the effect of using no cardiometabolic abnormalities as the reference group on the hazard ratio. The adjusted hazard ratio for having 3 or more abnormalities increased to 22.50 (95% CI: 11.21, 45.19) (Figure 1).

Abdominal obesity (HR = 2.81; 95% CI: 2.36, 3.33) and hyperglycemia (HR = 2.67; 95% CI: 2.26, 3.16) were the two strongest predictors of incident diabetes and were of approximately similar magnitude (Table 3). They were followed in order of decreasing magnitude by low high-density lipoprotein cholesterol, high blood pressure, and hypertriglyceridemia. The magnitude of the hazard ratio for the NCEP definition of abdominal obesity was larger among women than men (p interaction for abdominal obesity = 0.016), but the hazard ratio for the IDF definition of abdominal obesity was similar for men and women (p interaction for abdominal obesity = 0.370). Furthermore, the magnitude of the hazard ratio for hyperglycemia was higher among women than men (p interaction for hyperglycemia = 0.030). For the other three components, there was no evidence for a gender effect (p for all interactions terms >0.05).

**Table 3:** Adjusted hazard ratios (95% confidence interval) for incident diabetes by components of the metabolic syndrome among participants aged 35–65 years, European Prospective Investigation into Cancer and Nutrition-Potsdam Study 194–1998 to 2005

	**N**	**NCEP abdominal obesity**	**IDF abdominal obesity**	**Hypertriglyceridemia**	**Low high-density lipoprotein cholesterol**	**High blood pressure**	**Hyperglycemia**
**Total**	2796	2.81 (2.36, 3.33)	2.90 (2.29, 3.67)	1.26 (1.06, 1.51)	1.97 (1.64, 2.37)	1.52 (1.22, 1.90)	2.67 (2.26, 3.16)
**Men**	1186	2.44 (1.97, 3.02)	2.73 (2.02, 3.69)	1.20 (0.94, 1.52)	2.02 (1.58, 2.59)	1.95 (1.40, 2.72)	2.45 (1.97, 3.05)
**Women**	1610	3.62 (2.67, 4.90)	3. 05 (2.05, 4.52)	1.36 (1.04, 1.78)	1.95 (1.46, 2.61)	1.20 (0.89, 1.63)	3.21 (2.45, 4.19)

IDF = International Diabetes Federation; NCEP = National Cholesterol Education Program.

Adjusted for age, sex, educational status, smoking status, alcohol use, occupational activity, physical activity, concentrations of total cholesterol and C-reactive protein, and other components of the metabolic syndrome.

We also examined whether the metabolic syndrome provided prediction beyond that of its components. In models that included the five components in dichotomized form, as well as the covariates, the adjusted hazard ratio for the NCEP definition was 1.13 (95% CI: 0.84, 1.52) (p Wald chi-square = 0.418) and that for the IDF definition was 1.28 (95% CI: 0.94, 1.76) (p Wald chi-square = 0.123).

## Discussion

In this large prospective study, the metabolic syndrome was a strong predictor of incident diabetes. The NCEP but not the IDF definition of the metabolic syndrome proved to be a stronger predictor among women than men. Particularly, noteworthy was the high risk of developing diabetes when participants with the metabolic syndrome were compared with those who had no cardiometabolic abnormalities.

Our estimates of relative risk for the IDF definition were in line with those from other prospective studies that have examined the associations between the metabolic syndrome and incident diabetes. Most previous studies reported measures of relative risk ranging from 2.05 to 10.5 [5-13]. In comparison, the hazard ratio for all participants included in the analyses in the EPIC-Potsdam study was 4.59.

Few studies have examined the gender-specific risks for incident diabetes associated with the metabolic syndrome [22,23]. In the Beijing Project, the magnitude of the relative risks was higher among men than women for all four definitions used in that study [22]. However, it was not clear whether the gender differences were statistically significant. In the Framingham Offspring Study, the relative risk among men was 6.92 (95% CI: 4.47–10.81) and that among women was 6.90 (95% CI: 4.35–10.94) [23]. In contrast, we found that women had a significantly higher hazard ratio when we used the NCEP definition but had a similar hazard ratio when we used the IDF definition. This difference likely emanates from one or both of the two key differences between the definitions. The first major difference is that, of the five components that are included in the two definitions, abdominal obesity is the only one that is defined differently. We did find a significant gender interaction with abdominal obesity for the NCEP definition but not the IDF definition. The second important difference is that the IDF definition requires the presence of abdominal obesity in contrast to the NCEP definition, which weights all components equally. Additional studies are needed to get a clearer picture of potential gender differences in the risk for incident diabetes associated with the metabolic syndrome.

The high hazard ratio for diabetes when we contrasted participants with 3 or more cardiometabolic abnormalities with those who had no abnormalities is consistent with results from previous studies. In the West of Scotland Coronary Prevention Study, men with 4 or more abnormalities had a hazard ration of 24.4 (95% CI: 7.53–79.6) [24]. In the Framingham Offspring Study, odds ratios for those with 3 or more cardiometabolic abnormalities were 23.83 (95% CI: 5.80–98.01) among men and 29.69 (95% CI: 9.10–96.85) among women [23]. However, in an analysis of data from the British Regional Heart Study, participants with 3 abnormalities had a hazard ratio of 4.56 (95% CI: 2.48, 8.78) and participants with 4 or 5 abnormalities had a hazard ratio of 10.88 (95% CI: 5.77, 20.50) compared with participants who had no abnormalities [25]. In the Caerphilly Cohort Study, the relative risks associated were 7.05 for 3 abnormalities and 13.39 for 4 abnormalities [26].

In other studies, impaired fasting glucose was the abnormality most strongly associated with incident diabetes [8-11,22,23,27]. In the EPIC-Potsdam study, however, the hazard ratio for abdominal obesity was slightly higher than that for impaired fasting glucose.

In general, the few studies that have investigated the issue of whether the metabolic syndrome adds additional prediction for incident diabetes once its components are accounted for have concluded that this is not the case. However, only the WHO definition in men added additional prediction in a study conducted in Mauritius, and only the EGIR definition in men added additional prediction in the AusDiab [10,11]. In the EPIC-Potsdam study, the metabolic syndrome was also not a significant predictor of incident diabetes once its components were taken into account.

The hazard ratios for the two definitions were similar in our study. Given the high concordance in classifying participants as having or not having the syndrome in our study population, this is perhaps not too surprising. Most of the previous studies have found slightly higher estimates of relative risk for the NCEP definition than the IDF definition [5-13]. However, our calculations of the summary relative risks yielded estimates of a similar magnitude. Thus, the two definitions performed approximately similarly in predicting risk of new-onset diabetes.

Our study is subject to several limitations. First, incident diabetes was defined on the basis of self-reported data that were confirmed by physicians. Thus, we failed to detect undiagnosed cases of diabetes. However, if the association between the metabolic syndrome and undiagnosed diabetes was similar to that of diagnosed diabetes than our hazard ratios should be accurate [28]. Second, measurements of concentrations of glucose and triglycerides were measured on both fasting and nonfasting participants. We adjusted the threshold for defining diabetes to reflect nonfasting status. However, the percentage of participants with hypertriglyceridemia was likely overestimated. However, the subanalysis conducted on fasting participants who had fasted yielded similar hazard ratios to those of the full sample. Third, we were unable to adjust for some potential confounders such as family history of diabetes.

Considerable controversy surrounds the metabolic syndrome. Criticism has been leveled at the syndrome in part because of the dichotomization of the variables that have been included in the definitions. Undoubtedly the act of dichotomization results in some loss of predictive information and likely leads to a hereto undetermined underestimation of risk. Until the time comes when better risk functions that incorporate the continuous nature of these variables are developed and accepted by the medical community or when diabetes risk scores are conclusively shown to outperform the metabolic syndrome in predicting risk, a relatively simple tool like the metabolic syndrome can serve a useful function.

In conclusion, the metabolic syndrome was a strong predictor of incident diabetes in the EPIC-Potsdam study. The expression of this risk was particularly pronounced when participants with the metabolic syndrome were contrasted with those who had no cardiometabolic abnormalities. These findings reinforce the point that maximal risk reduction for contracting diabetes can be achieved by maintaining all five components that are included in the NCEP and IDF definitions of the metabolic syndrome in the normal range.

## Abbreviations

EPIC: European Prospective Investigation into Cancer and Nutrition; IDF: International Diabetes Federation; NCEP: National Cholesterol Education Program.

## Competing interests

The authors declare that they have no competing interests.

## Authors' contributions

EF conceived the study, conducted the analyses, and prepared the manuscript. MS contributed to the analyses and to writing the manuscript. TP, MB, HJ, and HB revised the manuscript for intellectual content. MS and HB supervised scientific issues of the study. All authors read and approved the final manuscript.

EF analyzed the data and drafted the manuscript; MS supervised the analysis of the data and helped draft the manuscript; TP participated in the study design and reviewed the manuscript; MB participated in the study design and reviewed the manuscript; HJ participated in the study design and reviewed the manuscript; HB was a principal investigator of the study and helped draft the manuscript.

## Disclaimer

The findings and conclusions in this article are those of the authors and do not represent the official position of the Centers for Disease Control and Prevention.
